# 05 Methotrexate improve the foot functional impairment in juvenile idiopathic arthritis

**DOI:** 10.1093/rheumatology/keac496.001

**Published:** 2022-10-07

**Authors:** Yasmine Makhlouf, Hanene Lassoued Ferjani, Lobna Ben Ammar, Dorra B Nessib, Wafa triki, Kaouther Maatallah, Dhia Kaffel, Wafa Hamdi

**Affiliations:** Department of Rheumatology, Kassab Institute of Orthopedics, Tunis, Tunisia

## Abstract

**Background:**

Foot involvement in juvenile idiopathic arthritis (JIA) is common and affects about 60% of children [1]. Often overlooked by patients and practitioners, ankle and foot disability has been poorly studied and there are no accepted clinical practice guidelines for the diagnostic approach, as well as for the therapeutic management.

**Objectives:**

Our study aimed to evaluate the effect of medical treatments on functional disability related to foot involvement in JIA.

**Methods:**

We conducted a cross-sectional study including patients with JIA according to the revised ILAR criteria, collected from the rheumatology department of the Kassab National Institute. Sociodemographic and disease data (activity assessed by the Juvenile Arthritis Disease Activity Score 10 (JADAS-10) and therapeutic modalities) were recorded. Ankle and foot involvement were investigated by questioning and physical examination. We used the Oxford ankle foot questionnaire for children (OxAFQ-C); a validated, simple and reproducible score to assess the impact on the quality of life of children with foot problems [2]. This questionnaire encompasses 14 items corresponding to three dimensions: physical, school and play, and emotional. Higher scores represent better functioning. We searched for an association between the different drug treatments for JIA and the OxAFQ-C score.

**Results:**

The study included 23 patients. The mean age was 13 ± 4 [6–18] years. The sex ratio was 0.42 with a female predominance. The age of the disease was 49 ± 40 months [6–180 months]. The distribution of the different forms of JIA was as follows: oligoarticular (*n* = 7), enthesitis-related arthritis (*n* = 6), polyarticular FR + (*n* = 1), polyarticular FR- (*n* = 3), psoriatic arthritis (*n* = 3), systemic (*n* = 1) and undifferentiated (*n* = 1). Pain on walking and limitation of the talocrural joint was found in 39% and 30% respectively. The mean JADAS-10 score was 6.72 ± 6.1 [0–20]. Ten patients had high activity. Eighteen patients (78%) were taking level I analgesics and 14 patients (61%) were on non-steroidal anti-inflammatory drugs, 12 of them on demand. Naproxen was the most commonly used drug, followed by diclofenac. Eleven patients (48%) were on a disease-modifying anti-rheumatic drug (csDMARD). Methotrexate (MTX) was prescribed in 30% of cases with a mean dose of 7.91 mg/week [7.5–10]. Only one patient was on sulfasalazine. Four patients (17%) were treated with biologics: Etanercept (*n* = 3) and Tocilizumab (*n* = 1). The mean scores of the different domains of (OxAFQ-C) were as follows: 73.52 ± 35.89 [0—100] in the physical domain, 84.2 ± 30 [6.25—100] in the school and play domain, 88.75 ± 24.71 [12.5 – 100] in the emotional domain. There was no statistically significant association between the different domains of the Oxford score and the use of analgesics or NSAIDs (*p*> 0.05). Similarly, there was no statistically significant association between the different domains of the Oxford score and treatment with biotherapy (*p*> 0.05). However, patients on MTX had less functional impairment of the feet with a significant improvement in the physical domain (99.26 on MTX *vs* 62.5 without MTX, *p* = 0.02, r = 0.6).

**Conclusion:**

Our work showed that only methotrexate was associated with an improvement in functional foot outcomes in JIA. Further studies are needed to highlight the effect of other therapies, especially biologics.

**References**

[1] Arkell-Kautiainen M, Haapasaari J, Kautiainen H, Vilkkumaa I, Mälkiä E, Leirisalo-Repo M. Favourable social functioning and health-related quality of life of patients with JIA in early adulthood. Ann Rheum Dis. 2005; 64(6):875–880

[2] Morris C, Liabo K, Wright P and Fitzpatrick R. Development of the Oxford ankle foot questionnaire: finding out how children are affected by foot and ankle problems. Child: care and health development. 2007; 33(5): 559–68.

The implication to policy, practice, research and advocacy

More studies are needed regarding the effect of therapeutics on foot involvement in JIA children

